# Extraction and characterization of anthocyanin pigments from Iris flowers and metal complex formation

**DOI:** 10.1016/j.heliyon.2024.e31795

**Published:** 2024-05-23

**Authors:** Zaker Bahreini, Mohammad Abedi, Alireza Ashori, Ali Parach

**Affiliations:** Department of Chemical Technologies, Iranian Research Organization for Science and Technology (IROST), Tehran, Iran

**Keywords:** Violet Iris, Iridaceae, Anthocyanin-metal complex, Metalloanthocyanin, Herbal blue-green pigments

## Abstract

Exploring the chemical processes and factors influencing the stability of the blue color derived from anthocyanins is a crucial objective in agricultural and food chemistry research. The ability of these compounds to bind with metals could potentially stabilize anthocyanins extracted from plant-based foods or enable modifying their hues for application as natural food colorants. This study had two core objectives - first, to extract and identify the major anthocyanin pigments responsible for iris flower coloration. Second, to selectively complex purified iris anthocyanins with aluminum (Al^3+^) and copper (Cu^2+^) ions, probing the coordination chemistry underlying synthetic metalloanthocyanin formation. Fresh iris flowers were collected and anthocyanins extracted using an optimized acidic solution. After separation, anthocyanins were complexed with metals Al^3+^ and Cu^2+^ at pH 5–6 to understand better the evolution of blue and green colors in anthocyanin-metal chelates. Characterization of anthocyanins and their metal complexes utilized UV–visible spectrometry, colorimetry (L\* a\*b\* values), FTIR spectroscopy, and LC-MS. Metal complexation of anthocyanins exhibited bathochromic shifts of visible absorption maxima from 538 to 584 nm for Al-complex and 538–700 nm for Cu-complex. Color changes were accompanied by decreased lightness (L\*, from 87 to 81) and color coefficients a\* (+5.4 to −6.8) and b\* (−12.2 to −4.8). LC-MS analysis identified five major anthocyanin aglycones: cyanidin (Cyd, *m*/*z* 289), delphinidin (Dpd, *m*/*z* 305), petunidin (Ptd, *m*/*z* 229), malvidin (Mv, *m*/*z* 329) and pelargonidin (*m*/*z* 273), along with various glycosylated derivatives. This work successfully isolated key iris anthocyanin pigments and elucidated their metal chelation interactions underlying expanded floral color production, bridging knowledge gaps about this underexplored genus.

## 1 Introduction

**Fig. 1:**
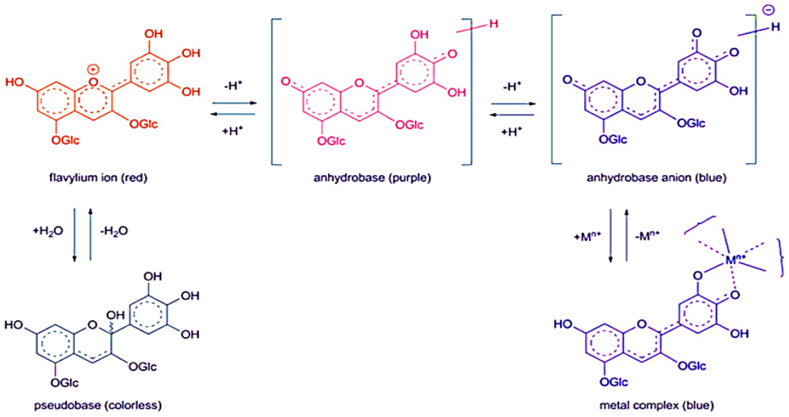
pH-dependent structural transformations of anthocyanins [5].

Plant pigments constitute a chemically diverse group of visually striking compounds that have garnered scientific interest for their aesthetic appeal and functional significance. These pigments impart a vast array of hues and structures that enable key physiological processes in plants. There are four primary pigment groups responsible for plant coloration: flavonoids and anthocyanins, betalains, chlorophylls, and carotenoids [1]. These compounds confer specific colors ranging from pale yellow to deep blue and dark red tones by absorbing distinct wavelengths of visible light. Beyond contributing color for visual function, plant pigments also play critical roles in resistance to stresses. Flavonoids and anthocyanins, in particular, protect plants from ultraviolet radiation damage, cold temperatures, and herbivores [2]. Their production of vivid red, purple, and blue flower pigmentation is incredibly vital. This coloration attracts pollinating insects as part of plants' reproductive processes. Overall, across their diverse structures and roles, plant pigments showcase elegant coordination between chemical form and critical ecological function. Their colors signify integral processes that have sparked lasting scientific curiosity. As a subgroup of polyphenolic flavonoids, anthocyanins make up the largest group of water-soluble pigments in the plant kingdom. They are responsible for producing the eye-catching red, purple and blue hues that color many fruits, vegetables, grains and flowers [3]. Over 500 distinct anthocyanins and 23 anthocyanidins have been identified to date. The backbone structure of anthocyanidins consists of an aromatic ring bonded to an oxygen-containing heterocyclic ring called the flavylium ion, which enables visible light absorption from 500 to 550 nm [1]. Attached is another aromatic ring with various hydroxyl, methoxy, and hydrogen substituents. Additional sugar residues then link to the aglycone anthocyanidins to form the glycosylated anthocyanin pigments (Fig. 1). The multitude of possible substituent combinations gives rise to the structural diversity observed among hundreds of anthocyanin pigments [1]. However, the conjugated system within the flavylium ion core drives the vibrant hues and light-absorbing properties that make anthocyanins essential for plant coloration while conferring a moderately astringent flavor profile [2,4]. The intriguing chemistry underlying their color and function continues to captivate researchers.

The structure and color of anthocyanins demonstrate remarkable dependence on pH. In highly acidic conditions below pH 2, the flavylium cation (AH^+^) form with vibrant red pigmentation prevails [6,7]. As pH increases above 2, reversible structural transformations occur, including hydration of the flavylium cation to generate a colorless hemiketal (B). This can further tautomerize into cis or *trans*-chalcone forms (Fig. 1) [5].

**Fig. 2:**
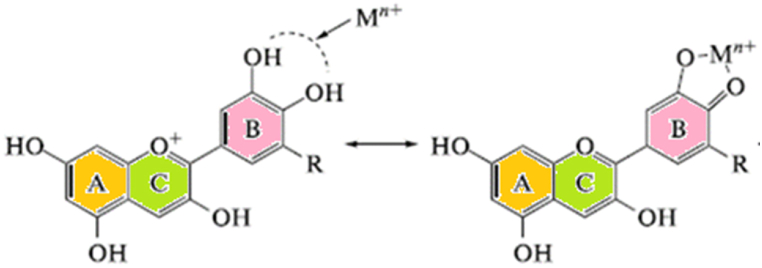
General structure of the anthocyanin-metal complex [6].

Loss of a proton converts the flavylium cation into a neutral purple quinoidal base. Finally, under more alkaline conditions between pH 6–8, the anionic blue quinoidal base structure (A^−^) becomes dominant. Coordination with metal ions forms complexes that stabilize the anthocyanin structure shifting the equilibrium to favor the bluer anthocyanin forms (Fig. 2) [8]. The specific substituents present on the B-ring, pH, tendency to form complexes with metals or other pigments, and metal binding interactions all influence the final vibrant color displayed by anthocyanins [9]. This multifaceted dependence illustrates the intricately sensitive relationship between anthocyanin structure and color expression.

Six major anthocyanidins - cyanidin, malvidin, peonidin, delphinidin, pelargonidin, and petunidin - account for the aglycone cores of over 80 % of all anthocyanin pigments in colored plant tissues [2]. Cyanidin derivatives occur most prevalently, representing 50 % of all anthocyanins in fruits and vegetables [1]. Pelargonidin, peonidin, and delphinidin constitute around 12 % each, while malvidin and petunidin occur less frequently at 7 % each [4]. Though many plants have been investigated for anthocyanin content, few studies have explored these pigments in Irises [[10], [11], [12]]. As the largest genus in the Iridaceae family with 300 species, Irises produce a rainbow of floral colors and represent an intriguing target for understanding anthocyanins' role in pigmentation [13,14]. The extensive color spectrum spans from Iris' popular ornamental blooms to applications in cosmetics, foods and pharmaceuticals [15]. This broad utility relates directly to anthocyanin's ability to impart vibrant hues as the primary floral pigment modulating Iris coloration [1]. Some metalloanthocyanin complexes also influence color diversity, with several metals reportedly complexing with anthocyanins to alter pigment color, notably toward blue hues [16]. Further research on Iris anthocyanins and metal complexes would expand the understanding of this intricate chemistry underlying their visually stunning floral palette. Complexation of anthocyanins with various metal ions can induce bathochromic shifts, resulting in metalloanthocyanins with altered hues. While multivalent metals like Al^3+^, Ga^3+^, Cr^3+^, Fe^3+^, and Mg^2+^ can yield bluer pigments, iron has been shown to degrade particular anthocyanins into brown tones in some berries [17]. Recent work demonstrates that *o*-dihydroxylated anthocyanins chelating metals at pH 5 are essential for generating blue colors in plants [18]. Analyses of blue hydrangea sepals revealed stable complexation between delphinidin derivatives and aluminum over a wide pH range in acidic ethanol solutions [19,20]. In comparing metalloanthocyanin stability, Buchweitz et al. [8] found Al^3+^-delphinidin-3-rutinoside chelates from eggplant more resilient to heat versus light exposure than acylated cyanidins in red cabbage. The degradation rate of delphinidin-aluminum complexes exhibits concentration dependence, with declining losses occurring over time, aligning with other recent studies. This exemplifies the delicate balance governing metalloanthocyanin pigment stability through subtle structural and environmental changes.

While anthocyanins have been widely studied in various plant sources, their characterization and metal complexation in ornamental flowers like irises has been relatively limited. The Iris genus, comprising over 300 species within the Iridaceae family, is renowned for its captivating floral displays showcasing a striking diversity of colors. However, detailed investigations into the specific anthocyanin profiles responsible for this vibrant coloration and their ability to form metalloanthocyanin complexes in irises are scarce. The objective of this study was to investigate two main research questions: first, to identify the primary anthocyanin pigments that contribute to the vibrant floral coloration in irises. Second, to examine whether the iris anthocyanins are capable of forming stable coordination complexes with metal ions, such as aluminum (Al^3+^) and copper (Cu^2+^), and if so, to characterize the consequent color changes and structural modifications. These metals were applied as model systems to probe the coordination chemistry governing the formation of synthetic metalloanthocyanin complexes from Iris anthocyanins. Monitoring color development in the Iris anthocyanin-metal solutions provided critical insights into the chelation interactions that modulate natural blue and green plant pigment production through similar metalloanthocyanin complexes. Achieving these dual aims expanded the current understanding of both the key pigments present in Irises and the specific mechanisms by which they interact with metals to generate expanded floral color diversity important for plant function. By focusing on this underexplored area, our work provides novel insights into the fundamental mechanisms governing the remarkable spectrum of hues observed in iris blooms, bridging the knowledge gap in this genus of immense ornamental and ecological significance.

## 2 Experimental

### 2.1 Materials

To ensure high quality and purity in the experimental procedures, we obtained HPLC-grade acetonitrile, trifluoroacetic acid (TFA) from Sigma-Aldrich (USA). These chemicals, including acetic acid, aluminum chloride hexahydrate, and copper sulfate, were purchased from Merck (Germany).

**Fig. 3:**
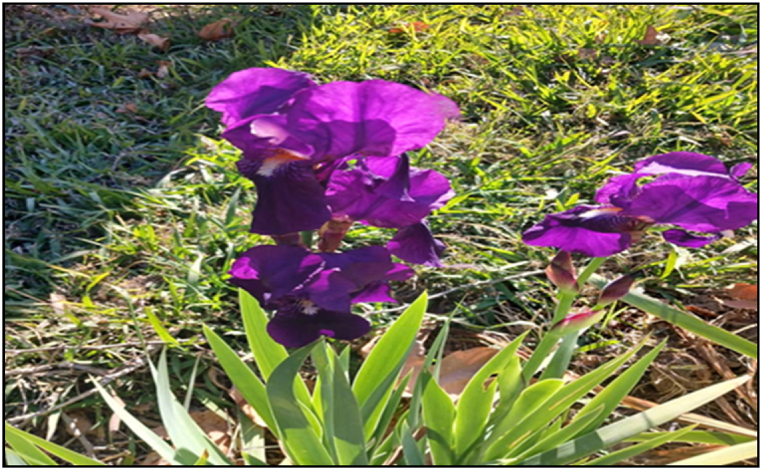
Iris flower used for anthocyanin extraction.

For sample collection, we carefully handpicked fresh violet petals from Iris flowers growing on the picturesque campus of the Iranian Research Organization for Science and Technology (IROST) (Fig. 3). This location was chosen due to its diverse and well-maintained floral ecosystem, ensuring a representative sample of Iris petals for our research. By gathering the petals on-site, we minimized the risk of any potential alterations or contamination during transportation.

### 2.2 Sample preparation and anthocyanin extraction

Before extraction, the Iris flower petals were rinsed with deionized water to remove any dirt or debris and then gently patted dry with paper towels. The petals were then chopped into small pieces of approximately 1–2 cm in size to increase the surface area for extraction. For the extraction, a sample to solvent ratio of 1:10 (w/v) was used, with 10 g of chopped Iris petals for every 100 mL of 0.1 % acetic acid solution in water. The extraction was carried out at a temperature of 50 °C for 2 h with continuous stirring. The extraction continued until the extracts became colorless, indicating that the anthocyanins had been effectively extracted [21]. To remove any solid particles, filtration through Whatman No. 1 filter paper and subsequent washing with water were carried out until a clear purple solution with a pH of 4.0 was obtained. To concentrate the anthocyanin solution, a vacuum rotary evaporation technique was employed, reducing the volume to approximately 100 mL. This step allowed for the removal of excess solvent and the concentration of the anthocyanins for subsequent analyses. To further purify the isolated anthocyanins, we utilized Sephadex LH-20 column chromatography. The crude anthocyanin extract was first loaded onto a glass column (2.5 × 60 cm) packed with Sephadex LH-20 resin and pre-equilibrated with deionized water. Isocratic elution was then carried out using deionized water as the mobile phase at a flow rate of 1 mL/min. Fractions of 5 mL were collected and monitored at 520 nm for the presence of anthocyanin pigments. The colored fractions were pooled together to obtain a purified mixture of the major anthocyanins present in the Iris petals. No further separation of individual anthocyanin components was performed at this stage. After the purification process, what was obtained was an enriched fraction containing a mixture of the major anthocyanin components from the Iris petals. The purification step aimed to remove impurities, proteins, sugars, and other compounds co-extracted during the initial solvent extraction process. However, the resulting purified fraction still consisted of a combination of the various anthocyanin pigments naturally occurring in the Iris flowers.

### 2.3 Synthesis of metal complexes

To prepare the metal complexes, the purified anthocyanins were first diluted using distilled water. The pH of the solution was then adjusted to 5.0, ensuring optimal conditions for the complex formation [22]. Metal salt solutions were prepared separately by dissolving aluminum (Al^3+^) or copper (Cu^2+^) salts in distilled water. The concentrations of the metal salts were adjusted to 0.5 M. Next, the metal ion solutions were added dropwise to the diluted anthocyanin solution. Upon adding the metal salts, a visible color change occurred, resulting in the formation of blue and green metal complexes. This color change indicates the successful binding of the metal ions to the anthocyanin molecules, forming stable complexes [5]. The formation of these metal complexes provides insight into the interactions between the anthocyanins and the metal ions, which can have implications for various applications, such as colorimetric sensing or metal ion sequestration.

### 2.4 Analytical methods

#### 2.4.1 UV-VIS spectrophotometry

To analyze the optical properties of the extracted Iris flower anthocyanins and the synthesized metal complexes, UV–visible spectra were recorded using a Lambda 25 PerkinElmer spectrophotometer (USA). The spectra were recorded over a wavelength range of 400–700 nm in 1 cm quartz cuvettes. UV–Vis spectra were obtained for the following samples.

1) Purified Iris anthocyanin extract in aqueous solution at natural pH (pH 4.5)
2) Purified Iris anthocyanin extract in acidified aqueous solution (pH 3.0)
3) Purified Iris anthocyanin solution after complexation with Al^3+^ at pH 5.0
4) Purified Iris anthocyanin solution after complexation with Cu^2+^ at pH 5.0

The acidic anthocyanin solution was prepared by adjusting the pH of the purified extract to 3.0 using 0.1 M HCl. For metal complexation, the pH of the purified anthocyanin extract was adjusted to 5.0 using 0.1 M sodium acetate buffer before adding the Al^3+^ and Cu^2+^ solutions, respectively. All solutions were diluted appropriately with deionized water to obtain absorbance readings within the linear range of the spectrophotometer. This range was chosen to cover the visible region of the electromagnetic spectrum, where the absorption and reflection of light by the samples can reveal valuable information about their molecular structure and electronic transitions. These spectral measurements serve as a crucial tool for characterizing the optical behavior of the samples and understanding the interactions between the anthocyanin molecules and the metal ions in the synthesized complexes.

#### 2.4.2 Colorimetry

The extracted anthocyanin solutions and synthetic metal complexes were subjected to analysis using a TES-135A TSE colorimeter. The colorimeter was specifically configured with illuminant D65, a 10° observer angle, and total transmittance settings. Before the measurements, the colorimeter was calibrated against a standard white plate to ensure accurate and reliable color readings. The color measurements were conducted using the CIE L*a*b* color system. In this system, L* represents the lightness of the sample and ranges from 0 (black) to 100 (white). The a* value indicates the presence of green hues when negative and red/magenta tones when positive. On the other hand, the b* value signifies the presence of blue hues when negative and yellow tones when positive. This analysis provides valuable information about the color properties, including lightness and hue, of the samples, enabling a deeper understanding of their visual appearance and potential applications.

#### 2.4.3 LC-MS spectrometry

To characterize and identify the anthocyanins from Iris flowers and their metal complexes, LC-MS analysis was performed. The analysis used a Waters C18 reversed-phase column with dimensions of 250 x 4.6 mm. The mobile phase consisted of two components: mobile phase A, which was a mixture of acetonitrile and 0.1 % formic acid, and mobile phase B, which was composed of water and 0.1 % formic acid. During the analysis, the column temperature was maintained at 35 °C, and a flow rate of 0.3 mL/min was employed. A 10 μL injection volume was used for each sample. The gradient protocol for elution was as follows: the mobile phase composition started at 20 % B and increased to 40 % B over 30 min. Subsequently, the mobile phase B composition was further increased to 50 % from 40 to 50 min and held for 10 min before returning to the initial composition of 20 % B. The detection of the separated compounds was carried out using a detector capable of performing full spectral scans from 190 to 800 nm. Precisely, anthocyanins were monitored at a wavelength of 520 nm, which is a characteristic absorption wavelength for these compounds. For mass spectrometry analysis, a Micromass Quattro micro-API instrument was employed. The nebulizer temperature was set at 350 °C, and a concurrent flow of 10 psi of nitrogen gas aided in sample nebulization. The capillary and cone voltage were set at 4.0 kV and 35 V, respectively. A drying gas flow rate of 200 L/h was utilized. During the mass spectrometry analysis, precursor ion masses and fragment loss reactions were evaluated to provide further information about the structure and composition of the anthocyanins and their metal complexes. By combining LC-MS analysis with spectral monitoring and mass spectrometry techniques, a comprehensive characterization and identification of the anthocyanins from Iris flowers and their metal complexes can be achieved. These analytical methods allow for the determination of molecular structures, fragmentation patterns, and other essential features, aiding in the understanding of the compounds' chemical properties and potential applications [23].

#### 2.4.4 Fourier transform infrared spectroscopy (FTIT)

FTIR was utilized to characterize the IR spectra of Iris flower anthocyanin and its metal chelates with Al(III) and Cu(II). The spectra were obtained at room temperature using a Broker Tensor 27 FTIR spectrophotometer over 4000 to 400 cm^−1^.

## 3 Results and discussion

### 3.1 UV–vis absorption spectra

**Fig. 4:**
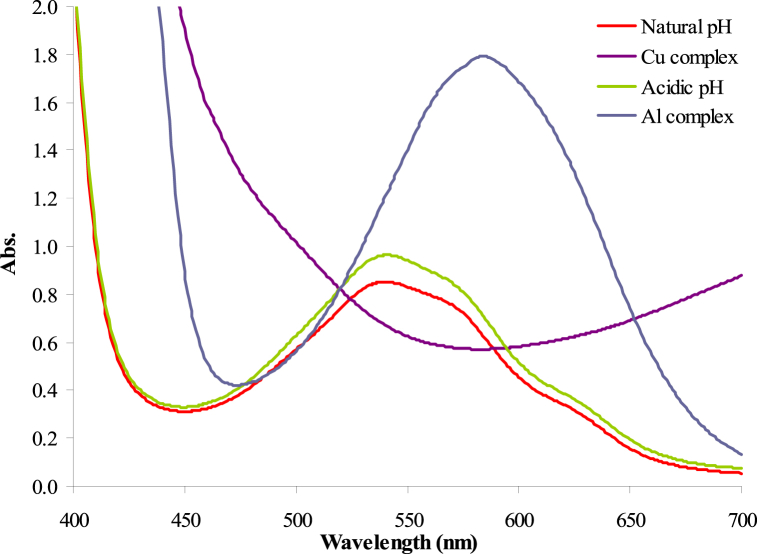
UV–Vis absorption spectra of Iris anthocyanins and metal complexes, natural pH, acidic pH, Al^3+^ complex, and Cu^2+^ complex.

Fig. 4 presents the UV–Vis absorption spectra of the extracted Iris anthocyanins at natural pH (pH = 4.5) and acidic pH (pH = 3.0) along with the spectra following complexation with Al^3+^ and Cu^2+^ metal ions. The Iris anthocyanin extract exhibits an absorption maximum wavelength (*λ*_max_) at 538 nm, responsible for its purple coloration. This aligns with previous floral anthocyanin analysis showing *λ*_max_ between 490 and 550 nm, corresponding to purple and red hues from the pigments conjugated flavylium cations [18,24,25]. Specifically, Iris pallida extracts were reported to have a similar *λ*_max_ at 535 nm. Complexation with Al^3+^ induced a substantial 46 nm bathochromic shift of the Iris anthocyanin *λ*_max_ to 584 nm. The Cu^2+^ complex demonstrates the most significant peak shift to wavelength larger than 700 nm, corresponding to more than ∼160 nm bathochromatic shift relative to the original anthocyanins. Prior work on Cu^2+^ complexes with cyanidin-3-glucoside from black rice also showed a similar bathochromatic displacement of 100 nm [26]. The bathochromic shifts stem from metal coordination stabilizing the quinoidal base structures of the anthocyanins. This enhances electron delocalization and lowers the energy required for visible light absorption. Comparable bathochromic shifts have been published for Al^3+^-chelated cyanidin-3-glucoside (58 nm shift) and Cu^2+^-complexed callistephin (80 nm shift) anthocyanins. The Al^3+^ chelation produced a blue color solution, while the Cu^2+^ complex resulted in a green hue [5,27]. The considerable absorption redshifts signify stable coordination complexes between the Iris anthocyanins and trivalent Al^3+^ and divalent Cu^2+^ metal ions. The differences in peak wavelengths likely arise from variations in the specific complex geometries, ligand-metal binding strengths, coordination sites occupied, and the resultant metal-anthocyanin stoichiometries for Al^3+^ versus Cu^2+^. The specific color development likely relates to the Iris anthocyanins' particular hydroxylation patterns influencing preferential metal binding sites. Overall, the observed spectral shifts and color changes are consistent with previous metalloanthocyanin analysis, confirming successful coordination complex formation between the Iris anthocyanins and Al^3+^/Cu^2+^ ions [28,29].

### 3.2 Colorimetry

**Fig. 5:**
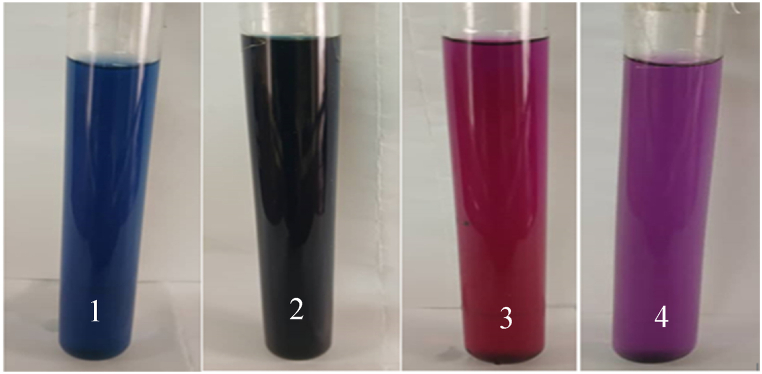
Photographs of Iris anthocyanin extracts and metal complexes Iris flower anthocyanins (ACN), (1) ACN-Al^3+^ complex, (2) ACN-Cu^2+^ complex, (3) Acidic ACN extract (pH 3), and (4) natural aqueous ACN extract (pH 4.5).

Table 1 shows the colorimetric data in the CIE L*a*b* color space for the extracted Iris flower anthocyanins and synthetic metal complexes with Al^3+^ and Cu^2+^. The anthocyanin extract has positive a* and b* values, indicating some redness and yellowness, which contributes to the purple coloration. This aligns with prior analysis placing purple tones in the red-blue range from positive a* and negative b* readings [30]. Complexation with Al^3+^ shifts the color parameters to lower lightness (L* 81.3), higher positive a* for residual red hues (+5.4), and negative b* (−12.2), denoting the blue components. Similar reductions in L* and b* values accompanied by slight decreases in a* have been reported for Al^3+^-complexed cyanidin derivatives, conferring bluer shades [27]. The Cu^2+^ complex resulted in the lowest lightness (L* 85.2), a negative a* value (−6.5) representing green hues, and a slightly negative b* (−4.8) for minor blue tones. These colorimetric coordinates match previous analyses where Cu^2+^ coordination with cyanidin glycosides produced greenish solutions, seen from lowered L* and negative a* readings around −5 to −10 [18]. Fig. 5 shows photos of the Iris flower anthocyanin extract at different pH levels (1–2), along with the Al^3+^ (3) and Cu^2+^ (4) complexes. The acidic extract is red, while natural pH produces purple, matching prior work on anthocyanin equilibrium shifts with pH influencing color [5]. The Al^3+^ and Cu^2+^ samples clearly show blue and green hues, respectively. Overall, the color changes arise from metal coordination altering the light absorption properties. The colorimetry and visual results confirm blue Al^3+^ and green Cu^2+^ metalloanthocyanin formation.

**Table 1:** Colorimetric data for Iris anthocyanins and metal complexes.

Compound	L*	a*	b*
ACN	+86.0	+14.0	+4.2
ACN-Al^3+^	+81.3	+5.4	−12.2
CAN-Cu^2+^	85.2	−6.5	−4.8

### 3.3 LC-MS spectrometry

**Fig. 6:**
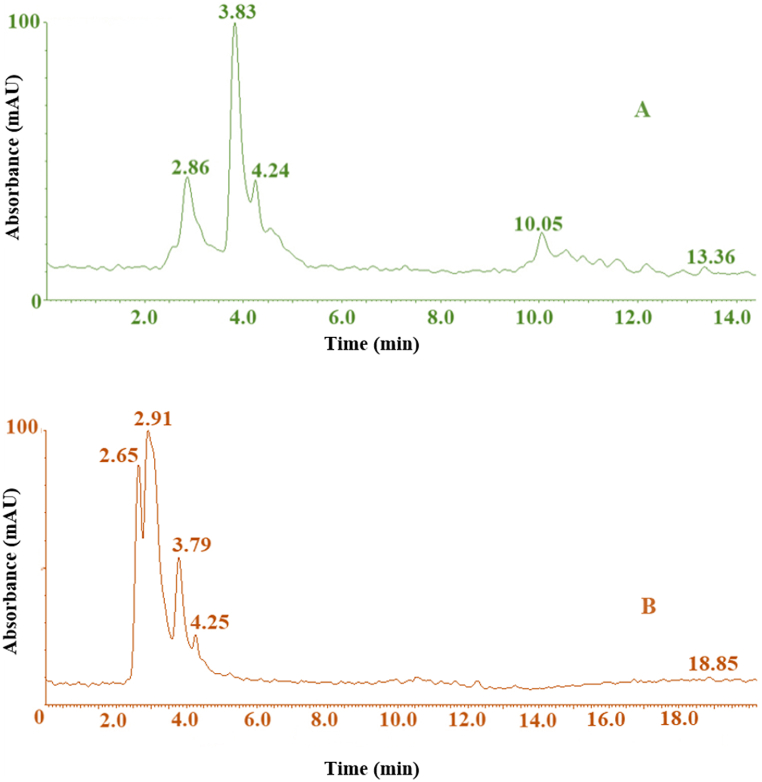
LC-mass spectrometry analysis of anthocyanin composition in Iris flower extract (A) and metal anthocyanin complexes (B).

Fig. 6A and Table 2 show the LC-MS analysis of the anthocyanin composition in the Iris flower extract. Five anthocyanin compounds were identified by their elution order and by comparing the *m*/*z* of each anthocyanin molecule and its fragmentation pattern to previous reports [10,11,31,32]. Peak 1 with *m*/*z* 578 and fragment ion 447 was identified as cyanidin-3-glucoside, consistent with other studies [10,11]. Peak 2 showed fragments at *m*/*z* 305, 204, 263, 513, and 232 indicating delphinidin derivatives [31]. Peak 3 had a molecular ion at *m*/*z* 607 (Cu^2+^ complex) and fragment ion at 229 and 273, suggesting a petunidin derivative [10]. Peak 4 exhibited [M]^+^ at 844 and fragments at 263, 273, 421, attributed to pelargonidin derivatives, the richest aglycone detected, aligned with previous work [11]. Peak 5, with *m*/*z* values of 321 and 329, is tentatively identified as a malvidin derivative, based on the fragment ion observed at *m*/*z* 287 [32]. In sum, five key aglycones were identified - cyanidin, delphinidin, petunidin, malvidin, and pelargonidin, the latter being the most abundant. Al^3+^ and Cu^2+^ complexation decreased or eliminated the prominent anthocyanin peaks with no observable metal complex fragments, although complex formation was evident through UV–vis, colorimetry, FTIR, and visual observation.

**Table 2:** Identification of major anthocyanins in Iris flower extract by LC-MS analysis.

Peak no.	*m*/*z* [M]+	Fragment ions (*m*/*z*)	Proposed identification
1	578	447	Cyanidin-3-glucoside
2	–	305, 204, 263, 513, 232	Delphinidin derivative
3	607	229, 273	Petunidin derivative
4	844	263, 273, 421	Pelargonidin derivative
5	321, 329	287	Malvidin derivative

Fig. 6B shows the LC-MS spectra following complexation with Al^3+^ and Cu^2+^, indicating metal coordination from the substantial peak decreases and shifts observed. This agrees with prior work noting intensity declines and retention time changes confirming metal chelation with anthocyanins from various plants [33,34]. Overall, the Iris anthocyanin structures detected closely match earlier floral pigment analysis. The spectral changes support successful anthocyanin-metal chelate formation through metal coordination reactions.

### 3.4 FTIR spectroscopy

**Fig. 7:**
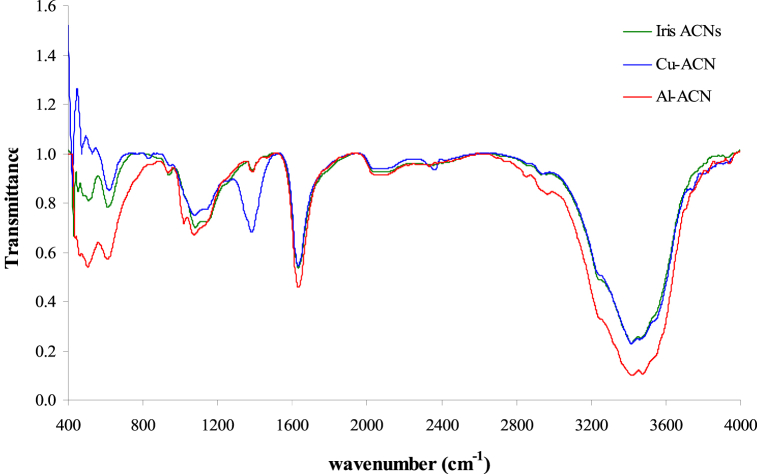
FTIR spectra illustrating metal coordination binding in Iris flower anthocyanin, anthocyanin-Al^+3^, and anthocyanin-Cu^+2^ complex.

Fig. 7 presents the FTIR spectra of the extracted Iris anthocyanins along with the Al^3+^ and Cu^2+^ metal complexes. The spectrum of the Iris anthocyanin extract displays characteristic peaks consistent with published IR data on anthocyanins [18]. The broad band at 3400 cm^−1^ corresponds to O–H stretching vibrations. The peaks at 2924 cm^−1^ and 2854 cm^−1^ signify C–H stretching of the anthocyanin structure. Additionally, the signal at 1655 cm^−1^ can be assigned to carbonyl C=O stretching vibrations. Upon complexation with Al^3+^, the IR spectrum exhibits shifts in several key signals. The O–H band at 3400 cm^−1^ decreases substantially, and the C=O vibration shifts to 1635 cm^−1^. Similar reductions in the O–H stretch coupled with a carbonyl peak shift to lower wavenumbers have been reported for Al^3+^ coordination with cyanidin-3-glucoside [27,28]. This provides evidence of aluminum binding to hydroxyl groups on the anthocyanin, as seen for the Iris anthocyanin-Al^3+^ complex. In the spectrum of the Iris anthocyanin-Cu^2+^ complex, the carbonyl vibration shifts further to 1627 cm^−1^. Additionally, new small peaks emerge at 610 cm^−1^ and 475 cm^−1^, indicative of Cu–O vibrations. Comparable carbonyl shifts and new Cu–O signals have been noted for Cu^2+^ chelation with delphinidin-3-sambubioside [29]. This confirms copper binding likely through the carbonyl and hydroxyl moieties for the Iris anthocyanin-Cu^2+^ complex. Overall, the FTIR spectral changes closely match literature reports on IR shifts and new peaks corresponding to metal coordination by anthocyanin hydroxyl and carbonyl groups. This provides strong evidence for aluminum and copper binding to the Iris anthocyanins, consistent with metalloanthocyanin formation.

## 4 Conclusions

The key findings of this work were the successful extraction and identification of major anthocyanin pigments from Iris flowers responsible for their vivid floral colors. Five anthocyanins were isolated and putatively identified via LC-MS spectrometry as cyanidin, delphinidin, petunidin/malvidin, and pelargonidin derivatives at various glycosylation states. Subsequent complexation with Al^3+^ and Cu^2+^ metal ions yielded hyperchromic effect and bathochromatic shifts of 44 nm and 160 nm, respectively, in UV–Vis absorption spectra, confirming stable coordination between the metals and Iris anthocyanins. Changes in color parameters and photographs visually validated the formation of blue Al^3+^- and green Cu^2+^-metalloanthocyanin complexes. FTIR spectral shifts specifically near 1060 cm^−1^ and 1500 cm^−1^ peaks further supported metal binding with hydroxyl groups on the anthocyanin structures. In summary, this work successfully isolated and identified key anthocyanin Iris pigments, elucidating their metal chelation interactions underlying the production of expanded blue and green floral hues. Further research can build on these fundamental findings to better understand, predict, and control the color modulation mechanisms across diverse anthocyanin-containing plants. Exploring chelation effects with additional multivalent metals beyond Al^3+^ and Cu^2+^ may offer routes to new vibrant plant pigment technologies and applications. Overall, this research advances insights into the delicate chemistry bridging anthocyanin structures with their intriguing optical properties.

## Data availability

Data will be made available on request from the corresponding authors.

## CRediT authorship contribution statement

**Zaker Bahreini:** Writing – original draft, Validation, Supervision, Methodology, Investigation, Formal analysis. **Mohammad Abedi:** Visualization, Validation, Methodology, Investigation, Formal analysis. **Alireza Ashori:** Writing – review & editing, Writing – original draft, Validation. **Ali Parach:** Investigation.

## Declaration of competing interest

The authors declare the following financial interests/personal relationships which may be considered as potential competing interests:

Alireza Ashori reports financial support was provided by 10.13039/501100017024Iranian Research Organization for Science and Technology. Alireza Ashori reports a relationship with Iranian Research Organization for Science and Technology that includes: employment. N/A If there are other authors, they declare that they have no known competing financial interests or personal relationships that could have appeared to influence the work reported in this paper.
